# Intestinal T-cell lymphoma not otherwise specified: a case report and literature review

**DOI:** 10.2144/fsoa-2023-0119

**Published:** 2024-05-20

**Authors:** Sarra Laabidi, Rabeb Hamouga, Sirine Bouslama, Rym Sellami, Soumoud Shimi, Asma Labidi, Jalel Boubaker

**Affiliations:** 1 Gastroenterology Department “A” La Rabta Hospital, Tunis, Tunisia; 2 Pathology Department Zaghouan Hospital, Zaghouan, Tunisia; 3 Dentistry Department Zaghouan Hospital, Zaghouan, Tunisia

**Keywords:** extranodal non-Hodgkin's lymphoma, intestinal T-cell lymphoma not otherwise specified, intestinal tumor

## Abstract

Intestinal T-cell lymphoma, not otherwise specified (ITCL, NOS), primarily affects the small bowel but can involve the stomach and large bowel. This report presents an uncommon case of ITCL, NOS in a patient affecting the large bowel, supported by a literature review. An 87-year-old female presented with abdominal pain, fever, vomiting and weight loss. Imaging revealed nodular thickening of the transverse and right colon, confirmed as polypoid mass lesions with ulceration through colonoscopy and biopsy, indicating ITCL, NOS. CT scan showed adrenal nodes classifying it as stage VI. The patient was referred for palliative care and passed away 40 days later, likely due to tumor progression. This case underscores the rarity of large bowel ITCL, NOS and the diagnosis challenge.

## Abstract (plain language summary)

Rare subtype lymphoma: intestinal T-cell lymphoma, not otherwise specified (ITCL, NOS), is an uncommon type of lymphoma that typically affects the small bowel but can also impact other parts of the digestive system.

This report presents a unique case in an 87-year-old patient where the tumor was found in the large bowel. Such occurrences are rare and pose diagnostic challenges due to atypical symptoms.

Diagnostic tools: imaging studies and biopsies are essential for diagnosing ITCL, NOS. In this case, abdominal imaging and colonoscopy revealed abnormal thickening and polypoid mass lesions in the large bowel.

Histological findings: biopsy results showed tumoral proliferation in the colon mucosa, with specific characteristics indicative of ITCL, NOS. Immunohistochemistry played a crucial role in confirming the diagnosis and should ruled out a variety of subtypes.

Advanced stage: CT scans revealed metastases to the adrenal glands and lymphadenopathy, classifying the disease as stage VI according to the Ann Arbor classification. This late-stage diagnosis often comes with a poor prognosis.

Extranodal non-Hodgkin's lymphoma frequently affects the GI tract, with B-cell lymphoma being more common than T-cell neoplasm [1]. Primary rectocolonic involvement accounts for only 0.1–0.5% of all primary GI lymphomas [2]. Intestinal T-cell lymphoma not otherwise specified (ITCL, NOS) is a rare subtype of enteric T-cell lymphoma characterized by a dense lymphoma infiltrate that can affect the small intestine, stomach, or large bowel. Clinical presentation of ITCL, NOS can vary, but common symptoms include weight loss, abdominal pain, and bowel obstruction, while less common symptoms include fever, diarrhea and anemia. Diagnosis is typically made using endoscopic biopsy, histological examination and immunohistochemical analysis. Treatment options for ITCL, NOS depend on the extent of the disease, with surgical resection being the preferred option for localized disease and systemic chemotherapy recommended for more advanced cases. The prognosis of ITCL, NOS is generally poor, with 5-year survival rate ranging from 30 to 40% [3]. In this article, we present a case of ITCL, NOS affecting the large bowel in an elderly woman and provide a review of the current literature on this rare lymphoma subtype.

## Case report

This case report describes an 87-year-old woman with a medical history of diabetes mellitus and hypertension who presented with abdominal pain, fever, vomiting and weight loss over a period of 2 weeks. Physical examination revealed paleness, cachexia and abdominal tenderness without palpable mass or lymphadenopathy. Skin examination was normal, without any suspected malignant lesions.

Laboratory tests revealed normochromic normocytic anemia with a hemoglobin level of 9 g/dl, leukocyte count of 10,310/μl, lymphocyte count of 910/μl, neutrophil count of 8770/μl, platelet count of 525,000/μl, creatinine of 106 μmol/l, lactate dehydrogenase of 470 UI/ml and C-reactive protein level of 31 mg/l. The blood smear result was normal and the bone marrow biopsy was not performed.

**Figure 1.**
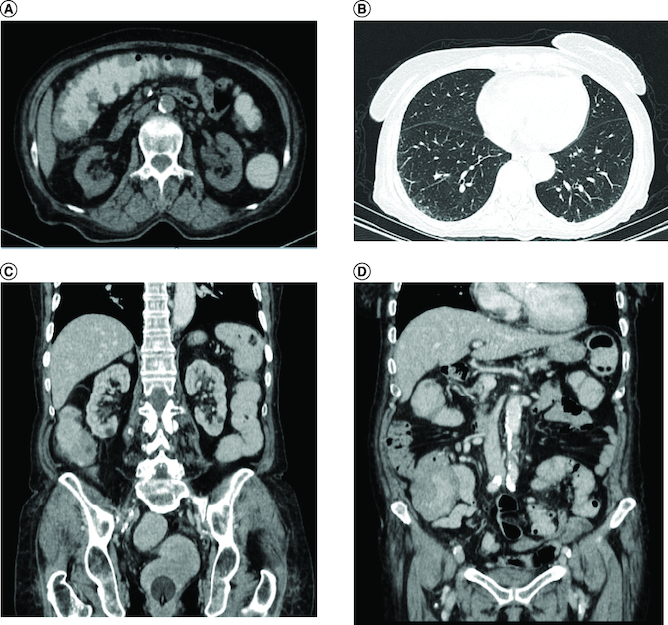
Coronal and axial scan. **(A)** Nodular thickening in the transverse and right colon, **(B)** adrenal nodes (23 × 31 mm) and latero cava lymphadenopathy, **(C)** thoracic CT scan shows no lymphadenopathy.

**Figure 2.**
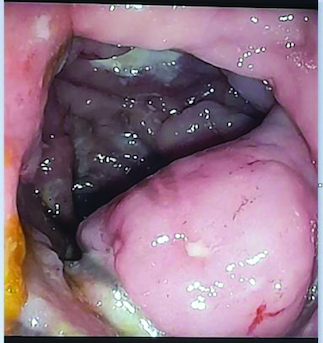
Polypoid mass lesion with ulceration.

**Figure 3.**
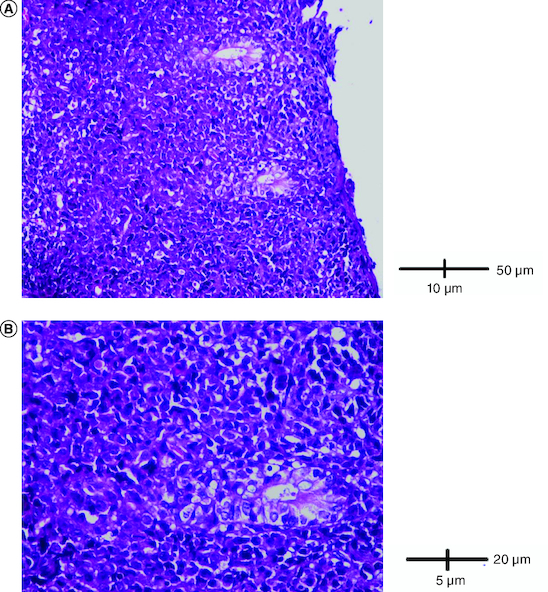
Histological examination. **(A)** A diffuse infiltrate of the colon mucosa that didn't invade crypts (HE × 200). **(B)** The infiltrate was composed of medium to large cells with atypical nuclei and numerous mitoses (HE × 400).

**Figure 4.**
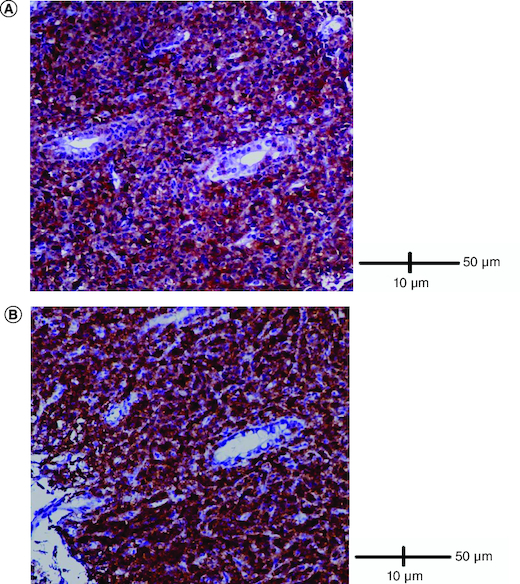
Immunohistological analysis. **(A)** Cells were diffusely positive for CD4 (IHC × 200). **(B)** Cells were diffusely positive for CD8 (IHC × 200).

**Figure 5.**
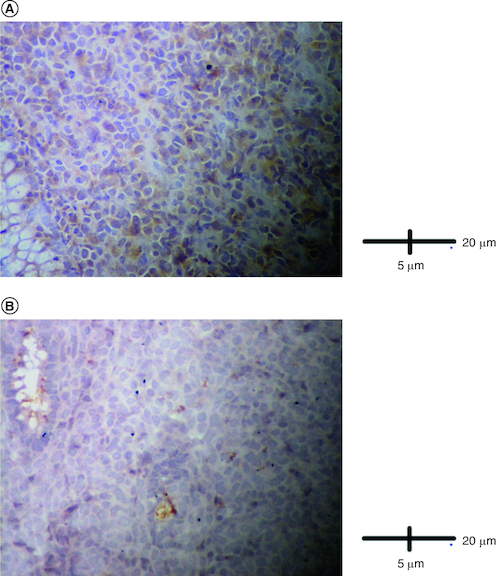
Immunohistological analysis. **(A)** Cells were negative for TdT (IHC × 400). **(B)** Cells were negative for ALK (IHC × 400).

Abdominal ultrasound and computed tomography revealed nodular thickening in the transverse and right colon with infiltration of pericolonic fat and a few subcentimeter lymph nodes (Figure 1). Colonoscopy detected polypoid mass lesions with ulceration ranging from 3 to 4 cm in size in the transverse and right colon (Figure 2). Histological examination of biopsies of these lesions revealed a diffuse infiltrate of the colon mucosa that did not invade the crypts (Figure 3). It is composed of medium to large cells with atypical nuclei and numerous mitoses. Immunohistochemical analysis showed that cells were negative for CK7, CK20, PS 100, Chromogranin A, synaptophysin, CD3, CD20, CD5, CD23, Bcl-2, Bcl-6, CD10, Pax-5, CD30, CD56, ALK, TdT, CD138 and CD1a. The cells were diffusely positive for CD4 and CD8 (Figures 4 & 5). The Ki-67 proliferation index was 100%. The patient was diagnosed with ITCL, NOS and further computed tomography scan revealed adrenal nodes (23 × 31 mm) and 14 mm long axis latero cava lymphadenopathy, classified as stage VI according to the Ann Arbor classification (Figure 1). The abdominal CT scan did not show any other deep lymphadenopathy. The thoracic CT scan did not show any lymphadenopathy and we did not proceed with a PET scan (Figure 1).

**Figure 6.**
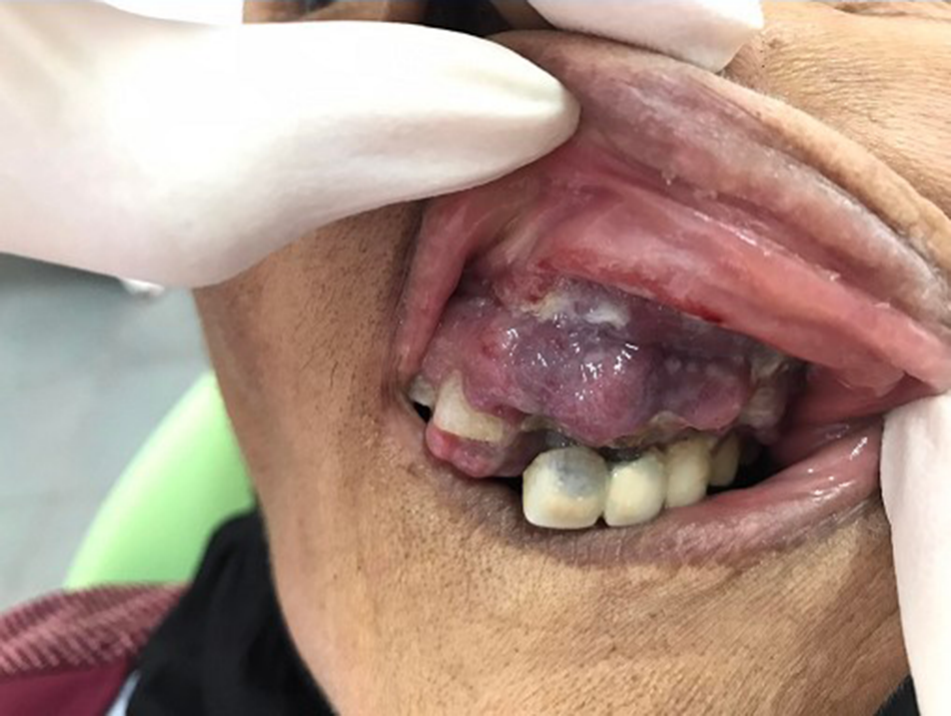
Ulcero-budding gum tumor with repression of the fixed prosthesis.

The patient was proposed to undergo the CHOP (cyclophosphamide, doxorubicin, vincristine and prednisone) protocol by the hematologist. Due to insufficient resources, HTLV-1 and coeliac serology as well as p53 mutations, EBER-ISH and TCL1 immunostaining were not performed. Few days later (15 days), the patient presented with an ulcero-budding gum tumor with repression of the fixed prosthesis (Figure 6). She did not undergo gum biopsy. She died 40 days after diagnosis, most likely due to tumor progression.

## Discussion

Primary intestinal T-cell lymphoma is an extremely rare condition and early diagnosis is often challenging [4]. In order to identify primary intestinal T-cell lymphoma, certain criteria must be met, including the absence of lymph node enlargement, normal blood counts, and presence of lesions primarily localized within the gastrointestinal tract [5]. The WHO classification 2017 defines four types of primary intestinal T-cell lymphoma : enteropathy associated t cell (EATL), monomorphic epitheliotropic intestinal T cell lymphoma (MEITL), intestinal T-cell lymphoma, not otherwise specified (ITCL,NOS) and indolent T-Cell Lymphoproliferative Disorder of the Gastrointestinal Tract (ITLPD-GI) [6,7]. EATL is usually associated with coeliac disease, is rare and aggressive, occurs primarily in the small intestine, and is composed of neoplastic cells that typically express CD3 [8]. MEITL, which has no association with coeliac disease, is derived from γδ and is typically positive for CD3, CD8 and CD56. ITLPD-GI is usually composed of CD8^+^ T cells with an indolent clinical course. ITCL, NOS, is a heterogeneous disease that affects the lymph nodes and extranodal sites. It is common in Asia and in the Caribbean region and typically arises in the large bowel or stomach. The clinical presentation consists of B symptoms, including fever, generalized lymphadenopathy, fatigue, and unexplained weight loss. The expression of TC antigens in ITCL, NOS, is variable, with no or reduced expression of CD5 and CD7. Loss of CD2 and CD3 is less common, and it can express CD8 and CD56. Other subsets show double positivity or double negativity for CD8 and CD4 [9].

Our case appears to be consistent with primary lymphoma ITCL, NOS, given the initial presentation with a large bowel tumor, absence of significant deep lymphadenopathy, and normal blood counts. Nonetheless, the likelihood of it being a systemic disease cannot be entirely ruled out. This is primarily because certain examinations, such as HTLV-1, coeliac serology and TCL1 immunostaining have not been conducted. Additionally, the presence of a gum tumor, even though it appears after the initial presentation remains a secondary factor to consider.

The patient's age does not align with coeliac disease, and in terms of immunohistological features, there are no compelling arguments in favor of another subtype.

In a literature review of 340 cases of peripheral T-cell lymphoma (PTCL), NOS, extranodal disease only was present in 13% of the patients. Common extranodal sites included the skin (16%), subcutaneous tissue (6%), and the lungs (8%) [10].

To the best of our knowledge, only four reported cases of ITCL, NOS affecting the intestine have been reported (Table 1).

**Table 1.** Case reports of intestinal T-cell lymphomas not otherwise specified.

Case	Age	Gender	Complication	Location	Treatment	Survival
1(12)	67	Male	Perforation	Small bowel	Surgery followed by chemotherapy	15 months
2(12)	65	Male	No	Small bowel	Palliative care only	13 months
3(13)	55	Male	Perforation	Small bowel	CHOP	
4(14)	73	Female	Perforation	Duodenum	Surgery	<2 months
Our case	87	Female	No	Colon	Palliative care	40 days

CHOP: Cyclophosphamide, doxorubicin hydrochloride [hydroxydaunorubicin], vincristine sulfate [Oncovin^®^] and prednisone.

Two cases were published in 2018 by a Chinese authors; the first one is about a 67 year-old man with a history of abdominal pain and without B symptoms (fever, night sweating and loss of weight). The tumor was localized in the small bowel and was complicated by perforation and lactate dehydrogenase was elevated. The patient underwent surgery followed by chemotherapy. The patient survived for 15 months. The second patient was a 65 year-old man with a history of abdominal pain, diarrhea, fever, night sweating and weight loss. The tumor was localized in the ileum and the patient received only palliative care. He died 13 months after diagnosis. Both patients presented ulcer, while only one had a focal necrosis in histological features. CD3, CD4 and CD5 were expressed in the two specimens, but CD8, CD56, TIA-1, Granzyme B and CD30 were not expressed in either of the specimen [11].

The following case described in 2022 in a 55 year-old man presented to the emergency with abdominal pain with a history of weight loss. A diagnosis of pneumoperitoneum by ileal perforation was retained. The final diagnosis of ITCL, NOS was rendered by immunohistochemistry, which showed positivity for CD3, CD4, CD8, CD103 and negativity for CD20, CD5 and CD56. The patient received CHOP (cyclophosphamide, doxorubicin hydrochloride [hydroxydaunorubicin], vincristine sulfate [Oncovin^®^], and prednisone) with improvement and no significant adverse out comes were reported after the treatment was started [12].

Finally, a 73 year-old woman with a history of coeliac disease presented for right upper quadrant pain reported by AzinMashayekhi in 2022 [13]. The tumor was complicated with a perforation; the neoplasic cells showed an annerant T-cell phenotype with coexpression of CD8 and absence of gene rearrangement. The patient survived less than 2 months after diagnosis.

In our case, the clinical presentation was similar to reported cases in the literature. The revealing symptoms can be subdivided into secondary signs to the tumor process (abdominal pain, diarrhea and intestinal obstruction), tissue destruction (intestinal perforation, peritonitis and rectal bleeding) and general signs (fever and weight loss). The colonoscopy revealed the presence of a polypoid mass lesion with ulceration in the right and transverse colon. This particular presentation raises suspicion of a mantle cell lymphoma, a rare condition characterized by the presence of multiple polypoid lesions affecting various segments of the digestive tract [14]. Generally, B-cell lymphomas tend to occur more frequently as fungating or ulcerofungating lesions, whereas T-cell lymphomas exhibit a higher prevalence of an ulcerative or ulceroinfiltrative pattern [15,16].

In the immunohistochemestry study, tumor cells were positive for CD8 and negative for CD5 which is common in the literature. The diagnosis of this entity may be confused with an inflammatory or infectious disorder. Therefore, a strong clinical suspicion of malignancy with negative histology should be not rule out the diagnosis and further repeated deep biopsies are strongly recommended during treatment and follow-up. If necessary, exploratory laparotomy may be indicated in early diagnoses. Numerous studies have attempted to identify clinically and pathologically significant features with prognostic implications in ITCL, NOS. However, the results of these studies have been inconclusive [17]. Recently, certain authors have developed an immunohistochemistry algorithm to distinguish between the two subtypes in paraffin-embedded tissue. This algorithm utilizes specific antibodies targeting key transcriptional factors (GATA3 and TBX21) and their corresponding target proteins (CCR4 and CXCR3) [18].

In our case the tumor was classified at stage IV and our patient died 40 days after the diagnosis. In a retrospective study involving 650 patients with newly diagnosed ITCLs and a median follow-up of 5.4 years, it was found that ITCL, NOS and other subtypes demonstrated lower 3-year conditional overall survival rates initially, along with higher risks of death (ranging from 26% to 44.3%). Furthermore, among patients who achieved complete remission following initial treatment, the prognosis varied depending on the specific histological subtype, with ITCL, NOS having a negative impact on the outcomes [19]. Nevertheless, common risk factors associated with survival were identified. These included poor Eastern Cooperative Oncology Group performance status (ECOG-PS), advanced stage of the disease, presence of extranodal involvement, bulky disease, elevated levels of lactate dehydrogenase and higher Ki67 proliferation rate. These factors were found to be significantly correlated with a shorter overall survival [20].

## Conclusion

ITCL,NOS is a rare subtype of intestinal lymphoma characterized by the presence of lymphoid cells that do not fit into any specific subtype. This case presents challenges in diagnosing primary intestinal lymphoma because of the rarity of this entity and the non specific endoscopic, radiological and pathological presentation and the literature review has identified existing case reports of ITCL, NOS, demonstrating the wide range of clinical presentations and outcomes. The prognosis is poor seen to later stages.
